# COVID-19, Framing and Naming a Pandemic: How What Is Not in a Disease Name May Be More Important than What Is

**DOI:** 10.3390/pathogens12020346

**Published:** 2023-02-18

**Authors:** T. S. Harvey

**Affiliations:** Department of Anthropology, Vanderbilt University, Nashville, TN 37240, USA; t.s.harvey@vanderbilt.edu

**Keywords:** COVID-19, disease naming, risk communication, public health

## Abstract

While the disease name and acronym COVID-19, where ‘CO’ refers to ‘corona’, ‘VI’ to virus, ‘D’ to disease, and ‘19′ the detection year, represents a rational, historically informed, and even culturally sensitive name choice by the World Health Organization, from the perspective of an ethnography of disease framing and naming, this study finds that it does not, however, readily communicate a public health message. This observation, based on linguistic and medical anthropological research and analyses, raises a critically important question: Can or should official disease names, beyond labeling medical conditions, also be designed to function as public health messages? As the ethnography of the term COVID-19 and its ‘framing’ demonstrates, using acronyms for disease names in public health can not only reduce their intelligibility but may also lower emerging public perceptions of risk, inadvertently, increasing the public’s vulnerability. This study argues that the ongoing messaging and communication challenges surrounding the framing of COVID-19 and its variants represent an important opportunity for public health to engage social science research on language and risk communication to critically rethink disease naming and framing and how what they are called can prefigure and inform the public’s uptake of science, understandings of risk, and the perceived importance of public health guidelines.

## 1. Disease Framing, When Similarities of Kind Get in the Way of Differences in Degree

### 1.1. The Basics

As late as February of 2021, on the Centers for Disease Control’s (CDC) website, under the caption, what is COVID-19?, ‘the basics’, which was written for the lay public, it stated:

A novel coronavirus is a new coronavirus that has not been previously identified. The virus causing coronavirus disease 2019 (COVID-19), is not the same as the coronaviruses that commonly circulate among humans and cause mild illness, like the common cold [1].

In the service of simplicity, by stating that “a novel coronavirus” is not the same as known (familiar) coronaviruses that cause mild illnesses such as the common cold, the CDC innocently compared considerably different things that share the same name (taxonomy), *coronavirus*, but that differed significantly (in degree) with respect to their potential impact on human health. For public health and risk perceptions in the United States (US), the consequence of a comparison of the *kind* that left the *degree* of the severity of the disease of COVID-19 unstated was, this paper argues, the subtle privileging of the known, the ‘common’, and the ‘mild’ illnesses over the (then) understated and potentially debilitating and deadly illnesses associated with the ‘novel’. In the early days of the pandemic, a period of roughly six months (February 2020 to July 2020), there was inadequate mention of the severe morbidities and potential mortalities associated with COVID-19 disease that made the CDC’s introductory comparison in “the basics” so consequential for informing emerging public perceptions of risk in the US [1]. As the analysis offered in this paper will suggest, these official omissions by the CDC, to borrow a term from James Gibson’s groundbreaking research in the psychology of perception [2,3], may have unwittingly “afforded” the lay public with a false comparison. That is, rather than working in the interest of public health, the CDC’s comparison helped to ‘frame’ national conversations about COVID-19 that associated it, time and again, with the mild illnesses of ‘common’ coronaviruses that cause colds.

The term ‘framing’, as it is used in this study, is based on research in sociology, communication studies, and cognitive science [4,5,6,7] and is broadly understood as the circulation of information presented to inform and influence social perceptions. More specifically and relevant to the topic of public perceptions of risk, framing involves ‘select[ing]’ some aspects of a perceived reality and making them more salient in a communication text, in such a way to promote a particular definition, causal interpretation, moral evaluation and/or treatment recommendation for the item described [8]. For COVID-19 public health messaging in the US, this study seeks to understand some of the complex ways in which the ‘framing’ of the disease in the public square may have been related to its official ‘naming’ by WHO, and how the two worked recursively to orient and inform emerging public perceptions of risk. As the analysis will demonstrate, disease framing, alongside naming, participates in meaning-making as important forms of knowledge production in public health emergencies. The study that follows is based on ethnographic, linguistic, and public health research conducted in person and online. It builds on “the ethnography of communication,” a foundational methodology in the field of linguistic anthropology developed by Dell Hymes [9] and applied here to a trans-disciplinarily study of public health communication and risk perception. The multimedia data collected from public demonstrations, articles, photographs, news interviews, social media, and videos represent public ways of speaking about and understanding COVID-19 in the US. Taken together, they provide an important snapshot of the pandemic and the role that language and communication played in framing and naming public health risks and perceptions.

**Figure 1:**
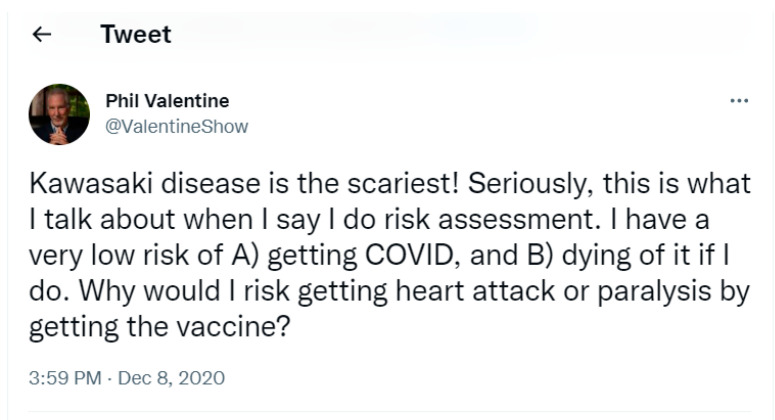
Screenshot of COVID-19-related tweet, dated 8 December 2020.

The following tweet, Figure 1, a pseudo-medical discussion of COVID-19, dated 8 December 2020 is an ethnographic example of what linguist George Lakoff identified as “metaphorical framing”. Here, the information is presented to influence public perceptions of risk by mapping the characteristics of one concept (Kawasaki disease) onto another (COVID-19) without the two actually being related [6,10,11,12].

Phil Valentine hosted a nationally-syndicated conservative talk radio show that aired weekdays out of Nashville, Tennessee, which, at its height, was broadcast on over 100 stations nationwide. The tweet, emblematic of metaphorical framing, is a comparison of dissimilar conditions presented to reduce public perceptions of the risk of COVID-19. The resulting juxtaposition of the ‘scariness’ of the exotic-sounding ‘Kawasaki’ disease, mucocutaneous lymph node syndrome, with the implied ‘ordinariness’ of COVID-19, is not only an example of misinformation [13] and xenophobia (discussed later in the paper) but ‘communicability’. That is, what the ethnographic research of Charles Briggs and Daniel Hallin defined as the “effects of power [that] emerge from everyday ideological constructions of how information is purportedly produced, circulated, and received, how individuals and institutions participate in this process, and how statements are infused with authority and value” [14]. 

For context and clarification, despite the visually disturbing physical effects that mucocutaneous lymph node syndrome can have on children, swelling in the walls of small to medium-sized blood vessels, inflammation of the coronary arteries, swelling in the glands and mucous membranes inside the mouth, nodes, eyes, and throat (Mayo Clinic), it is a treatable condition and largely considered non-contagious. By contrast, the physical effects of COVID-19 on the human body are far less visible, it is a highly contagious disease, and more importantly, at the time of the tweet, there were neither vaccines nor therapeutics available to treat it in the US. Early in the pandemic, Valentine became a vocal skeptic of the severity of COVID-19, slowly emerging as an outspoken national critic of COVID-19 vaccines and related public health guidelines and mandates. His tweet displays what this study identified as a common skeptical framing of COVID-19, the expression of an outsized (amplified) concern with the health impacts of taking the vaccine and a minimized (attenuated) concern for the potential impacts of contracting the disease and/or following public health guidelines. 

### 1.2. COVID-19 in the “Public Square”

**Figure 2:**
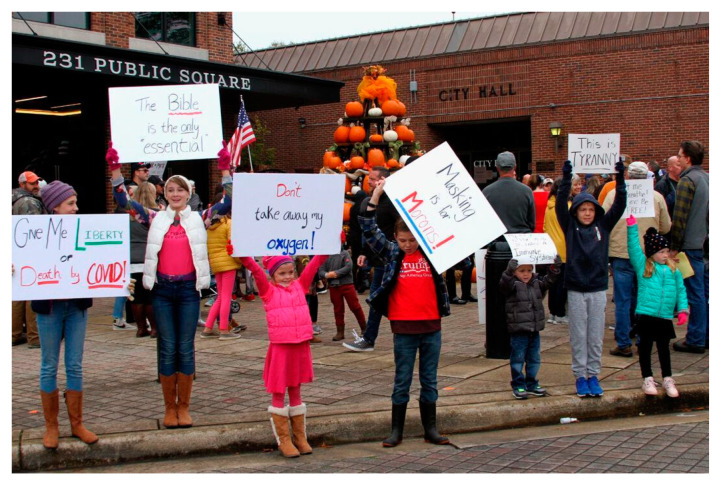
“We will not comply!” a protest against COVID-19 public health guidelines in middle Tennessee (Photograph, Carole Robinson, Williamson Herald 2020) [21].

Valentine’s views were not, however, unique to him, the ethnography shows that his comments should be situated within the wider narrative [15] patterns and practices within his community [16]. Consider the photograph below, Figure 2, from the Williamson Herald, showing signs held by children during one of many middle Tennessee protests against public health guidelines. In the foreground, left, one of poster reads, “Give Me Liberty or Death by COVID”, another, “Masking is for Morons”, and less visible, center right, “My Mask is on the Inside, it’s called an Immune System!” Publicly voicing and circulating attenuated views of the risk of COVID-19, Valentine once famously said that he believed that were he to become infected, his chances of dying would be, in his words, “way less than one percent”. Tragically, in July of 2021, Valentine, who was not vaccinated, contracted COVID-19 and died the following month, leaving behind a wife and three children. Before his untimely death, from a hospital bed suffering from COVID pneumonia, Valentine purportedly changed his position on vaccines, urging listeners and followers nationwide to “get the shot”. According to his brother Mark, at the end of his life, Phil expressed regret, saying that he believed that a lot of people didn’t take the vaccine because he didn’t take it. Understanding the potential health impacts of examples such as these and their disproportionate impacts on certain sectors of populations requires building on ethnographic research in the areas of health/communicative inequality and health news [17] as well as studies on the role of the media in risk communication [18,19,20].

**Figure 3:**
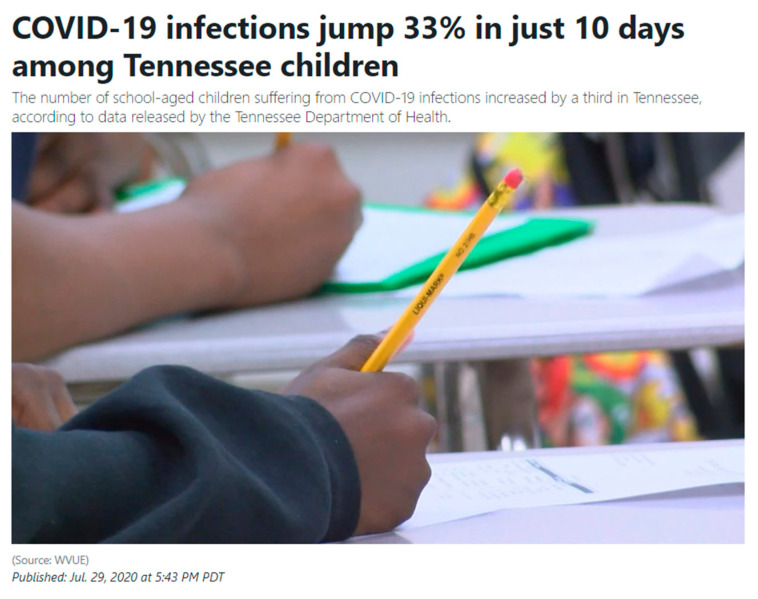
Screenshot of a local news report discussing the impending reopening of schools in Tennessee in the face of an alarming rise in infection rates (WTVF/WVLT, July 2020) [25].

As the ethnographic examples demonstrate, for public health, one downstream concern for de facto comparisons of dissimilar conditions such as COVID-19 and the common cold should be the action possibilities [2] that this kind of framing can afford. That is, the accumulating reduction in public perceptions of risk [22,23]. If left unchecked, widely-held low public perceptions of risk in the face of potentially harmful or life-threatening public health emergencies can become, in their own right, compounding collateral vulnerabilities [24]. Figure 3, a screenshot from News Channel 5, highlights the work that framing COVID-19 played in debates within the US over whether or not to reopen public schools in the fall of 2020. In July of the same year, in middle Tennessee, against a backdrop of COVID-19 vaccines not being available to children nationally and positive testing rates above 10% locally, official messaging on reopening schools regularly sought to reduce public perceptions of risk by weighing the potential health impacts of children becoming infected with the disease against the dissimilar (though significant) risk associated with children not attending to school in person. 

Despite some federal officials recommending that states avoid fully reopening schools in the fall of 2020 if the positivity test rates were above 10% and the Tennessee Department of Health documenting that “the number of school-aged children suffering from COVID-19 infections [had] increased by a third in Tennessee”, public schools fully-reopened. This study defined these kinds of biopolitical [26] social determinants of health [27,28] as “iatrogenic vulnerabilities”, those related to or are induced by ‘official’ public health messaging. Seemingly acknowledging the problems emerging from the popular and persistent conflation of COVID-19 with the common cold, Dr. Anthony Fauci, director of the National Institute of Health (NIH) and his colleagues coauthored an article in the *Journal of the American Medical Association* (*JAMA*) entitled, “Coronavirus Infections—More than Just the Common Cold”. Their paper explains that while scientists had long considered human coronaviruses (HCoVs) “as inconsequential pathogens, causing the ‘common cold’ in otherwise healthy people”, in the 2lst century alone, three “highly pathogenic HCoVs (SARS-CoV, MERS-CoV, and 2019-nCoV) have caused global epidemics with alarming morbidity and mortality” [29]. Despite efforts such as this, aimed primarily at medical audiences, nearly two years after the CDC’s initial framing of COVID-19 and the subsequent emergence of numerous variants, a piece published in The Atlantic, dated 15 December 2022, asks, “Is COVID a Common Cold Yet?” The article expresses a continued association of COVID with the common cold in the US and a cautious hope for a return to the normality that was the ordinariness of the common cold. The piece, when examined as a public way of speaking about COVID-19, raises an important question about time, disease, and risk. Specifically, what are the potential impacts of the official convention of dating diseases, for example, adding 19, the detection year, to COVID? Might this practice, downstream, years into a pandemic or epidemic, lead the public to consider a ‘dated’ disease and the risks that were associated with it as things of the past? 

## 2. Rethinking Uncertainty, Public Health Messaging between Risk and Vulnerability

If the CDC, following the World Health Organization (WHO), was committed to a framing of COVID-19 that associated it with common coronaviruses in the minds of the US public, the efficacy of which will be debated, what their public health messaging needed most, in the place of the simplicity that they offered in “the basics” was distinction—a combination of clarity and subtlety. That is, the CDC needed enough flexibility in messaging to acknowledge the complexity of COVID-19 and the gravity of a shifting public health crisis without either amplifying or attenuating the risk as the pandemic ebbed and flowed between pre-crisis, crisis, and post-crisis phases [30] of variants. Discursively, this is a difficult needle to thread, particularly in a crisis, where risk communication research shows that for the lay public, the authority of official discourses [31] is often linked, for good and ill, to the attributed durability of the claims in the face of changing situations. To navigate contingencies such as these, the WHO’s 2017 guide, “Communicating risk in public health emergencies”, recommends “communicating uncertainty”. Specifically, they note that “communication by authorities to the public should include explicit information about uncertainties associated with risks, events and interventions, and indicate what is known and not known at a given time” adding that “messages need to be reviewed and reshaped periodically as the emergency evolves” [32].

“We are certainly right now in this country out of the pandemic phase…”

Fauci, 4/27/22 NPR [33].

“It’s not over…” “We are in a different moment of the pandemic.”

Fauci, 4/28/22 APNews [34].

The above quotes by Dr. Anthony Fauci from a PBS NewsHour interview dated 27 April 2022 (cited by NPR), and the clarification that followed a day later in his interview with Associate Press, demonstrate the challenge that evolving situations such as pandemics present to public health messaging and the critical role that communicating uncertainty plays in helping the public to reduce their risk and vulnerability. In light of WHO guidelines on communicating risk, to the credit and detriment of the CDC, by largely steering clear of public health messages that emphasized the “novel” and “unknown” aspects of COVID-19, it successfully avoided what risk communication research on public health emergencies warns are “fright factors” for the public connected to uncertainty [18,23,35]. However, the downside of messaging strategies overly preoccupied with amplification (fright) [36] is the alternative, risk attenuation. That is, the potential, especially early on, for public health messaging to aid in the production of ‘publics’ (portions of populations) insufficiently concerned about the risk of infection and subsequently doubtful about the need for public health measures and mandates, e.g., masking, social distancing, and quarantining [37].

The COVID-19 pandemic has highlighted, among many things, the changing meanings and interpretations of public health messages, a topic perhaps nowhere more visible than in the proliferation and circulation of information, misinformation, and disinformation about the disease, in particular, on social media platforms [38,39,40]. With the awareness that information can spread faster than diseases has come a renewed interest in the importance of communication in public health emergencies. Recognizing the significance of the public uptake of reliable health information and its impacts on health, the WHO has labeled the current situation, a crisis in communication. At the Munich Security Conference on 15 February 2020, the current Director-General, Tedros Adhanom Ghebreyesus commented, “We’re not just fighting an epidemic; we’re fighting an infodemic” [41]. Defined as, “too much information including false or misleading information in digital and physical environments during a disease outbreak”, from the view of the WHO, an infodemic can cause “confusion and risk-taking behaviours that can harm health” and lead to mistrust in health authorities, undermining the effectiveness of public health responses [42]. 

The links that this paper draws between the relationship of public health messaging to public perceptions of risk, vulnerability, and the COVID-19 pandemic, are supported not only by sociolinguistic research but also by what the WHO notes is the quality of the evidence in the grey literature [43]. The argument for rethinking public health messaging (around the framing and naming of disease) is not based on the presumption that an outcome of such inquiries will be a simplistic one-size-fits-all recommendation of how to “get it right” or, on the other hand, the unrealistic expectation that it will ever be possible to completely immunize public health communication from misinterpretation, manipulation, or even deliberate disinformation. On the contrary, the study’s argument is that health “messages need to be reviewed and reshaped” as public health emergencies emerge and evolve, a position consistent with WHO guidance. The urgency of the current need to reconsider the framing and naming of disease exists not despite but in light of the problems of miscommunication and disinformation, and what we are now only beginning to learn about the impacts of the so-called ‘infodemic’ on public health [44]. 

## 3. Beyond Labeling, Disease Names as Important Frames for Public Health Messaging

As Toppenberg-Pejcic and her colleagues studying emergency risk communication point out, one important global response to the epidemics of Ebola, Zika, and Yellow Fever was the acknowledgment that in public health emergencies, in addition to the need for food, clothing, shelter, and safe drinking water, emergency risk communication is essential. Beyond the material necessities of life, “people also need to know how best to avoid risk so that further injury, morbidity, and mortality can be minimized” [45]. Consistent with this observation, in 2017, the WHO released its first-ever guidelines for emergency risk communication (ERC) and “communicating risk in public health emergencies”. Based on a grey literature review, their recommendations fall into three categories, (a) building trust and engaging with affected populations, (b) integrating ERC into health and emergency response systems, and (c) ERC practice. While the sociolinguistic concerns raised in this study around disease naming and public perceptions of risk are relevant to all three of the categories, the most direct implications are for the category of ‘messaging’. Under ERC practice recommendation, C4.1 states that “risk should not be explained in technical terms, as this is not helpful for promoting risk mitigation behaviours” [32]. Applying these perspectives to the study of official disease naming practices at the WHO, the analysis in this section asks, does the name COVID-19 communicate the health risk of the disease, and how well does (or did) it provide for the informational needs of the public?

For public health messaging, particularly in the first six months of the COVID-19 pandemic, it was precisely what made this novel virus different, its potential severity, that was and remains (as of this writing) most worthy of distinction and regrettably not stated under the CDC’s ‘the basics’. It is important to note, however, that the CDC’s framing of COVID-19, which associated it with the common cold, cannot be separated from the WHO’s official naming of the disease that preceded and informed it. For risk communication and reduction, the corona or crown-like appearance (i.e., the morphology) of the virus that causes COVID-19 is far less important for public health than the *degree* of its contagiousness and virulence or severity (Button 2010). To begin with the WHO’s choice of the acronym COVID-19, where ‘CO’ refers to ‘corona’, ‘VI’ to virus, ‘D’ to disease, and ‘19’ the detection year, while a rational, historically informed, and culturally sensitive name choice, from the view of sociolinguistics, the disease name does not, in-and-of-itself, readily communicate a public health message. When one considers that according to the WHO, the current form of disease naming is intended “to enable [public] discussion on disease prevention, spread, transmissibility, severity and treatment (2021)” this paper’s seeming straightforward linguistic observation raises a number of questions with public health implications. First, what, if anything, can broader social science research on acronyms and their intelligibility contribute to discussions of their use as disease names in public health? Second, would accepting the WHO’s current naming practices, which utilize technical acronyms for disease names and supplements them, as needed, with public health messages (from government organizations) aimed more at the lay public, address the communicational challenges of the current moment? Third, should official WHO naming practices and government organization framing practices (e.g., such as those of the CDC) be rethought or redesigned with the aim of reducing confusion and promoting understandings that can help the public reduce its risk, and avoid injury, morbidity, and mortality? If the answer to the last question is yes, then the naming of COVID-19 represents an important opportunity to critically rethink how the labeling of a disease can name, frame [46], and prefigure the public’s uptake of science and risk communication, particularly during public health emergencies [47]. Before offering an assessment of current disease naming practices at the WHO, a technical and scientific distinction must first be made between diseases and viruses.

ICTV announced “severe acute respiratory syndrome coronavirus 2 (SARS-CoV-2)” as the name of the new virus on 11 February 2020. This name was chosen because the virus is genetically related to the coronavirus responsible for the SARS outbreak of 2003. While related, the two viruses are different.

[42]

Unlike diseases, the viruses that cause them are officially named by the International Committee on Taxonomy of Viruses (ICTV) and, according to WHO guidelines, these official names are based on “their genetic structure to facilitate the development of diagnostic tests, vaccines and medicines” [42]. Social and cultural constructedness of these categories notwithstanding [48,49], official international virus names are largely designed for use in and by science and medicine. By contrast, as discussed earlier, the WHO names ‘diseases’ with public health in mind, their International Classification of Diseases (ICD) is designed to enable discussions on disease prevention, spread, transmissibility, severity, and treatment. By the WHO’s own assessment and distinction, what diseases are called matters for public health promotion, risk reduction, and prevention and it is by this measure that the disease name and the communication of COVID-19 are considered and assessed in this paper. 

From the perspective of medical linguistic anthropology, the framework employed here [50,51,52] one key concern in the naming of diseases, particularly in public health emergencies, should be “communicability” [53] That is, a recognition that understanding the “communicational role” of disease names requires consideration of “the power of ideologies of communication” [54] as well as attention to their “productive capacities” to not only inform individual perceptions of risk but also to produce the very ‘publics’ for whom public health messages are designed and directed [55]. To examine the productive capacities of COVID-19 as a disease name, this study adopts a transdisciplinary approach, borrowing from formal linguistics and specifically, pragmatics, and speech act theory. 

Beginning with the sociolinguistic observation that for recipients of public health messages as much their producers, part of the problem with using acronyms such as COVID-19 for disease names is that their use overlooks three basic considerations of effective communication: (1) the word’s recognition (its intelligibility), (2) the word’s meaning (its comprehensibility), and (3) the meanings that the producer(s) of the word intended (its interpretability). Here, an attunement to pragmatics (i.e., the relations between language and their users) provides a basic framework for rethinking disease names with an eye towards some of the underutilized yet critically important ‘functions’ of language relevant to communicating and mitigating risk during public health emergencies. Although linguists have long recognized what Roman Jakobson called the ‘functions of language’ and the fact that words do not simply or self-evidently convey information directly [56,56] or even indirectly [57], these taken-for-granted observations within linguistics remain specialized elsewhere and have not always translated well across disciplines. Not surprisingly, then, the functions of language have not informed official disease nomenclature guidelines and practices. Historically, the WHO has seen the challenge of naming diseases as a referential or a denotive problem and more recently, in response to critiques of xenophobic disease names, an indexical or associative problem. 

At its most basic, applying the framework of ‘speech act theory’ to the study of disease names and their role in public health [58] means understanding them not only as presenting or transmitting information but also as potentially performing actions in the world. Most relevant to the topic of public health emergencies and risk communication, speech act theory demonstrates that words (and by implication disease names) can function to ‘advise’, ‘warn’, and ‘assert’. Further, the approach argues that ‘interpretability’ or the public uptake of messages relates, at least in part, to the function of words [59,60]. In light of these insights, if a central aim of disease naming at the WHO (in contrast with virus naming) is to enable public discussions on prevention, spread, transmissibility, severity, and treatment, it might be useful to engage sociolinguistics and consider developing disease names with an intended ‘communicative function’ in mind (e.g., risk reduction). Such a shift would involve the WHO considering disease names (which currently also name outbreaks, epidemics, and pandemics) as not only suitable labels but also early and potentially critical public health messaging opportunities for “framing” public perceptions. 

## 4. How Acronyms Alienate Audiences, Overestimate Understanding, and Complicate Communication

We’ve been repeating a pattern. Now with these new omicron subvariants—I call them the Scrabble variants, because they’re these high-valued Scrabble letters such as Q and X and B. We’re seeing cases go up again (excerpt from Andrew Dansby’s interview with Peter Hotez, published 17 October 2022 in RE/New Houston).

**Figure 4:**
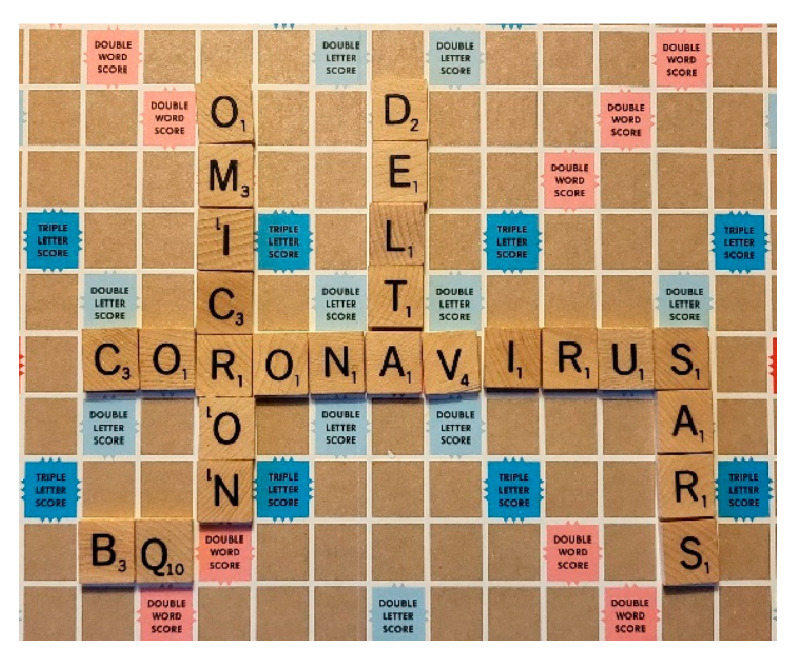
“Scabble Variants”, photograph and artwork by the author, December 2022.

In the above quote, Dr. Hotez, who is the dean of the National School of Tropical Medicine at Baylor College of Medicine and a globally recognized public health advocate, characterized COVID-19 subvariants as “Scrabble variants” such as B.Q.1.1. His comments, illustrated in Figure 4, highlight not only the ongoing communicational challenges that acronyms present to naming and framing disease for public health but also why the use of these terms should be reconsidered. Research on publications in the fields of psychology and environmental sciences, among others, demonstrates that the use of acronyms as discursive conventions, that is, as specific ways of speaking within specialized fields [61,62] can actually alienate audiences and overestimate their familiarity with an abbreviation [39,63,64,65,66,67] The most well-known cases of this (written and spoken) are the communicational problems created and associated with ‘medicalese’ and ‘legalese’ [68,69,70] in the fields of medicine and law, respectively. Taken together, a broad range of research on acronyms and jargon [71,72] shows, in large part, that their use reduces intelligibility, the opposite of the intent of public health messages. Yet, acronyms have become the primary way that the WHO names diseases and their variants, particularly since their revised naming policy was implemented in 2015. 

Importantly, the communicational problems of audience alienation and overestimation of comprehension are not unique to acronyms (e.g., NAGPRA and STEM), which in the strict technical sense, “only encompasses abbreviations that are pronounced as words” [65]. Interpretability is also reduced with the use of initialisms or the arrangements of letters (pronounced separately) that stand in for words (e.g., EPA, NATSB, HIV). The specialized terminologies of science expressed in abbreviations (both acronyms and initialisms) have also been shown to limit the reach of scientific knowledge within and across disciplines. Examining the relationship between jargon (which includes acronyms) and scholarly citations, researchers sampled over 21,000 articles on cave research [73], a notably interdisciplinary field, and found that the use of jargon in “the title and abstracts of articles significantly reduces the number of citations a paper receives” [74]. Yet, as important as these transdisciplinary findings [75,76,77] on the communicational problems associated with acronym use are, they are not presented here to simplistically suggest that abbreviated disease names should be replaced with lay or non-technical terms. Research on medical communication in this area demonstrates, not surprisingly, that the use of technical terms in medicine, when compared to the use of lay terms for the same ailments, are linked to public perceptions of the seriousness of health problems, conditions understood as rising to the level of ‘disease’ [78,79,80]. Put another way, simplicity in health communication can come at a cost, often the attenuation of public and patient perceptions of risk and the seriousness of disease [81]. 

Returning now to the specific use of the acronym COVID-19 for the disease and pandemic name, does or did it constitute jargon? That is, a complex concept shared by those, for example, in a common profession (virology, medicine, and public health) but one that precludes others without the same specialized knowledge from fully understanding? The answer would seem to be yes, particularly early on in the pandemic. Could an overestimation of the public’s familiarity with the seriousness of a disease, based on their apparent ‘fluency’ in and use of a technical term [82] such as COVID-19, help to explain some of the seemingly irrational disagreements that emerged during the pandemic, for example, over the contagiousness and severity of the disease or subsequent debates about public health guidelines such as masking and social distancing? Acronyms stand for something and in many cases, different things over time, a fundamental challenge for risk reduction and public health messaging becomes, therefore, understanding and addressing the distribution of those meanings over disparate spaces, times, contexts, and populations. 

The phrase, “covid is a cold virus”, which circulated in the public square within certain portions of the US population, illustrates a fundamental misunderstanding of the disease and a concrete example of the conflation of the kind of virus with the *degree* of severity. As discussed previously, addressing lay misunderstandings such as these was, in part, the aim of publications such as the one co-authored by Dr. Anthony Fauci in *JAMA*, “Coronavirus Infections—More than Just the Common Cold” [29]. While the statement “covid is a cold virus” was misleading and raised serious concerns about the dangers of attenuating the public’s perception of the risk of COVID-19, based on the CDC’s de facto comparison offered in ‘the basics’, the claim was not altogether false. More egregious, however, were the kinds of overtly biopolitical interpretations of the COVID-19 acronym circulating within the US during the early days of the pandemic. For example, public claims that COVID stood for the “Certificate of Vaccination Identification” by Artificial Intelligence or even more problematic and xenophobic, the “Chinese Originated Viral Infectious Disease”, an issue discussed later in the paper. These examples highlight some of the problems associated with using acronyms for disease names that, in addition to being poorly understood and inadequately defined, do not ‘frame’ the risk of the disease for public health. 

Findings from research on doctor–patient communication may also provide some relevant insights and guidelines for public health communication [83,84,85]. For example, studies on doctor–patient communication have long demonstrated that the use of medical terms (jargon) by patients in clinical encounters does not indicate that they share meanings and understandings with health professionals who use those same terms professionally. In fact, overestimation as with underestimation [86] of patient or lay public familiarity with medical terms can contribute to misunderstandings [51,81]. Taken together these wider observations on medical communication seem to suggest that perhaps the WHO might consider revising or even jettisoning their use of acronyms (science jargon) in favor of names that can both label a disease and potentially function as public health messages (e.g., warning, advising, asserting). If not acronyms for disease names, then what? Why the way back to the historical use of proper nouns for disease names is not likely the path forward to communicating future risk.

## 5. The Lingering and Malignant Place of Prejudice in Public Health Messaging

There is a long history to the practice of using non-acronym names for diseases, outbreaks, and epidemics. In recent years the WHO and others have found the results of decades of taken-for-granted disease naming practices involving the use of proper nouns (in particular) to be deeply concerning and in some cases, injurious to the peoples, places, and things named. Here, one need only consider a few of the voluminous examples that could be cited. The Spanish Flu of 1918 (H1N1) that was not actually Spanish (the first case was identified in Kansas), Hong Kong Flu (China), West Nile Virus (Uganda), MERS (Middle East Respiratory Syndrome), Ebola Hemorrhagic Fever (named after a river in the Democratic Republic of Congo), Mexican Swine Flu, Avian Flu, Zika Virus (named after a forest in Uganda) [87], and Monkey Pox (named after outbreaks in colonies of monkeys kept for research but frequently associated with African rodents and monkeys). As problematic as these examples of ‘naming and framing’ are, particularly in the matter-of-fact way in which these official (legitimating) discourses associate the so-called third world, its peoples, cultures, and places with disease and uncleanliness [88], to only point out the xenophobia and ethnocentrism inherent in these historical practices does not go far enough. 

**Figure 5:**
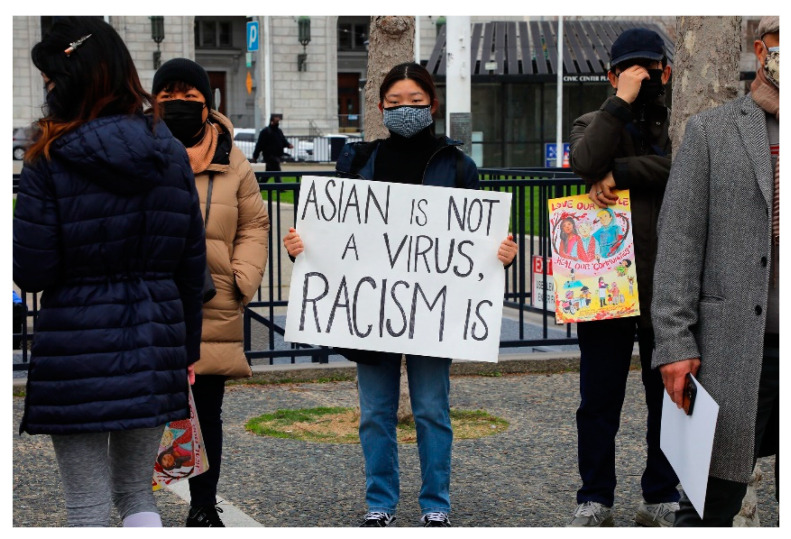
“Asian is not a Virus, Racism Is”, photograph by Jim Wilson, New York Times, 2 March 2021 [101].

The WHO, in its landmark 2015 guide, *Best Practices for the Naming of New Human Infectious Diseases* acknowledges these problems and aims, in its words “to minimize unnecessary negative impact of disease names on trade, travel, tourism or animal welfare, and avoid causing offence to any cultural, social, national, regional, professional or ethnic groups” [89]. According to the guide, from the date of its publication onward, official disease names may not include and should avoid: using geographical locations (e.g., cities, countries, regions, continents), people’s names (e.g., Creutzfedt–Jakob disease, Chagas disease), species/class of animals, or foods (e.g., swine flu, bird flu, monkey pox), cultural, population, industry, or occupations references (e.g., legionnaires, miners, butchers), or terms that incite undue fear (e.g., unknown, death, fatal, epidemic) [90]. While these guidelines are an important start, it is necessary to also consider some of the difficult questions for public health and risk perception that have resulted from decades of these systemic practices and more importantly, the lingering publics and malignant perceptions that these racializing discourses [91,92] have informed and helped to create. Xenophobic disease names and the policies and practices that produce and circulate them [26,93] demonstrate the history of the place of prejudice in public health [94,95,96,97]. Regrettably, public health messages along with the ‘publics’ that they produced [37]. form parts of contemporary dialogs and as such are not remnants of some ‘dark’ or forgotten past [98,99]. On the contrary, historic xenophobic disease names are “linked to current and future uses” [100] (see, for example, Figure 5) that reverberate into the present with potential impacts on perceptions, public health, and even human safety.

According to findings from the Center for the Study of Hate and Extremism in 2020 Anti-Asian hate crimes in large US cities increased by 149% and, according to the Federal Bureau of Investigation, 76% nationwide. Without suggesting or implying that correlation equals causation, these figures can be placed in a larger sociolinguistic context of COVID-19 in the US where some political discourses consistently framed the disease as the ‘China Virus’ and K’ung-Flu’, [102] well before the origins of the disease were known, which (as of this writing) are still being investigated. Xenophobic disease names such as these not only use but leverage some of the same functions of language (e.g., ‘asserting’) that this study has suggested might be of useful to public health messaging. But to what purpose? This is a complicated and difficult question to answer, particularly given that there may well be no single satisfactory answer. Could the strategic use of xenophobia in contemporary public health messaging in the US, as problematic and racist as it is, somehow be understood as useful? Not likely, unless its premeditated use is deployed as a kind of marketing ‘avoidance motivation’, utilizing fear (‘fright factors’), to direct [49,59] publics (‘sanitary citizens’) away from the supposed ‘dangers’ of disease and towards adherence to official guidelines and mandates? Another nearly unspeakable but not unthinkable question is, could the purpose be to produce anti-Asian sentiments? If so, how would such sentiments be in the interest of public health outside of creating scapegoats, peoples, and places, on whom the blame for viral outbreaks such as COVID-19 can be placed [103], sifting the shared global responsibility away from multinational corporations (e.g., agribusinesses) where “the most dangerous new diseases in humans can be traced back to food systems” [24]

**Figure 6:**
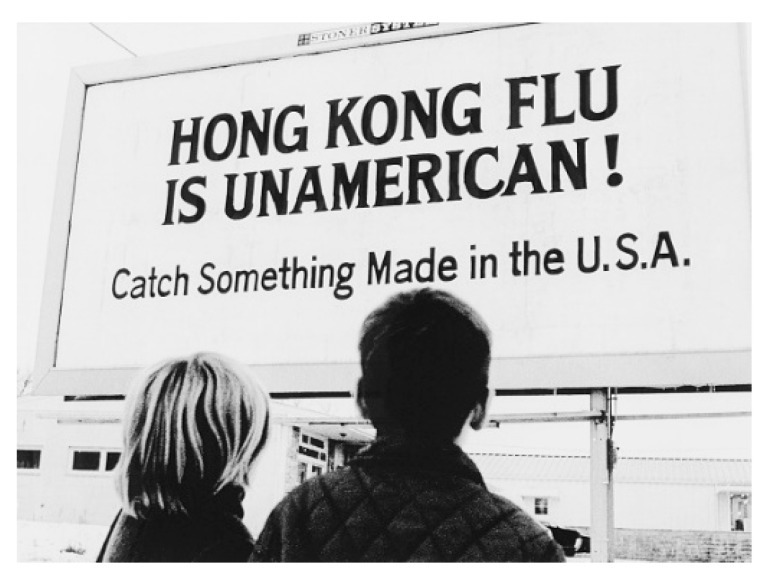
“Hong Kong Flu is Unamerican! Catch Something Made in the U.S.A.“, billboard advertisement, Des Moines, Iowa, 1968, photograph Bettmann Archive, Getty images [109].

Xenophobic disease names, the nativist public health policies that they articulate, and the historical associations that they make are reminiscent of reoccurring and malignant troupes [104], a condition that Ralph Trouillot’s famously diagnosed as ‘Savage Slot” [58]. Such framing does not merely intimate, it declares the superiority of the US (as the West), attributing inherent inferiority to Asia, the perennial ‘sick man’ and elsewhere other, portrayed reprehensibly as the archetypes of unsanitary subjects and backwardness. Discourses like those in Figure 6. have long conflated certain racialized peoples and places with diseases [105]. More disturbing still, systemically, disease names and frames such as these give a place to prejudice, xenophobia, and bigotry squarely inside of public health [106]. They produce, as they circulate, ‘publics’, who are encouraged to follow health guidelines based not only on the risk that a particular disease might pose or personal precaution but rather out of fear of racialized and demonized others [107,108] who have been blamed historically for all matter of disease and misfortune.

## 6. Considering Disease Names That Can Frame Public Health Messages 

Problems with acronyms notwithstanding, from the perspective of risk communication, ironically, the ICTV’s official name for the virus, SARS-CoV-2, in its unabbreviated form, “severe acute respiratory syndrome coronavirus 2”, does a much better job, from a sociolinguistic perspective, communicating the disease’s potential health risk to the public than the now ubiquitously used name, COVID-19. It does this despite the fact that the words ‘acute’ and ‘syndrome’ may themselves be technical terms or jargon in some communities, as discussed previously. Arguably, disease names such as “severe acute respiratory syndrome”, among others, convey public health messages, in part, because they read and sound like phrases, groups of words functioning as meaningful units. As a public health message, the function of the name, severe acute respiratory syndrome, labels the disease while communicating its virulence (‘severe’ and ‘acute’), warning and alerting the public about the potential symptoms and bodily systems affected (‘respiratory syndrome’). Despite being imperfect and not addressing all the unique communicational challenges presented by this disease, specifically, those who are infected but who remain asymptomatic, the current virus name does nonetheless put the severity of the most likely and consequential symptom, acute respiratory syndrome (for those who are symptomatic), front, center, and top of mind for the public. There are other disease names in their unabbreviated form, the inclusion of technical disease terminologies notwithstanding, that function better as public health messages than their acronym counterparts. Consider, for example, Sexually Transmitted Disease (STD), Chronic Obstructive Pulmonary Disease (COPD), Urinary Tract Infection (UTI), Restless Leg Syndrome (RLS), Erectile Dysfunction (ED), and Post Traumatic Stress Disorder (PTSD), to name a few. 

Taking the disease name of COVID-19 as its focus, the medical and linguistic anthropological analysis offered in this paper argues that if the goal of the WHO and the CDC, particularly during public health emergencies, is to reduce the risk and spread of a disease and combat the misinformation, what diseases are officially called matters a great deal. A central contribution of this paper’s analysis of the framing and naming of diseases and its examination of some of the problems associated with acronyms is the argument that disease names should be conceived and designed to be comprehensible, intelligible, and interpretable to the wider public. Beyond labeling, disease names can perform the critically important function of framing public health messages. 

Further social science research in risk communication will be needed to determine if (or to what degree) adopting disease names that also function as public health messages can help address some of the risk perception and attenuation problems associated with acronym disease names such as COVID-19. Until additional data is available, the implications of this study’s analysis of current disease naming practices at the WHO, while potentially significant, are not a recommendation. Further, this preliminary sociolinguistic analysis cautions against institutions and organizations seeing a potential change to official disease naming or framing as a simple or ‘silver bullet’ solution. While certain aspects of what disease names convey to the public remain relatively stable (e.g., the classification of COVID-19 as a severe acute respiratory syndrome), other, more variable, features of public health messaging will continue to necessitate change and be supplemented around whatever ‘fixed’ disease names are used as epidemics, pandemics, and the needs of the public unfold. Moving forward, whatever changes to disease naming and framing that may come in the future as a result of sociolinguistic research, disease naming will need to continue to adhere, per the WHO’s 2015 guidelines, to the moral obligation of avoiding the use of proper names that associate diseases, pandemics, epidemics, and outbreaks with peoples, cultures, places, and animals. Disease names can frame public health messages that inform and advise, reducing risk by conveying reliable information that supports public discussions of severity, spread, prevention, and treatment.

## Data Availability

The data presented in this study is contained within the article.
